# A Bayesian optimization tunning integrated multi-stacking classifier framework for the prediction of radiodermatitis from 4D-CT of patients underwent breast cancer radiotherapy

**DOI:** 10.3389/fonc.2023.1152020

**Published:** 2023-06-13

**Authors:** Kuan Wu, Xiaoyan Miu, Hui Wang, Xiadong Li

**Affiliations:** ^1^ Department of Tumor Radiotherapy, The First People’s Hospital of Fuyang Hangzhou, Hangzhou, China; ^2^ Department of Tumor Radiotherapy, Hangzhou Cancer Hospital, Hangzhou, China; ^3^ Key Laboratory of Clinical Cancer Pharmacology and Toxicology Research of Zhejiang Province, Affiliated Hangzhou First People’s Hospital, Zhejiang University School of Medicine, Hangzhou, China; ^4^ Department of Radiotherapy, Affiliated Hangzhou Cancer Hospital, Zhejiang University School of Medicine, Hangzhou, China; ^5^ Laboratory of Medical Imaging and Translational Medicine, Hangzhou Cancer Hospital Affiliated to Zhejiang University School of Medicine, Hangzhou, China

**Keywords:** breast cancer, radiation therapy, radiation-induced dermatitis, radiomics, stackingclassifier

## Abstract

**Purpose:**

In this study, we aimed to develop a novel Bayesian optimization based multi-stacking deep learning platform for the prediction of radiation-induced dermatitis (grade ≥ two) (RD 2+) before radiotherapy, by using multi-region dose-gradient-related radiomics features extracted from pre-treatment planning four-dimensional computed tomography (4D-CT) images, as well as clinical and dosimetric characteristics of breast cancer patients who underwent radiotherapy.

**Materials and methods:**

The study retrospectively included 214 patients with breast cancer who received radiotherapy after breast surgeries. Six regions of interest (ROIs) were delineated based on three PTV dose -gradient-related and three skin dose-gradient-related parameters (i.e., isodose). A total of 4309 radiomics features extracted from these six ROIs, as well as clinical and dosimetric characteristics, were used to train and validate the prediction model using nine mainstream deep machine learning algorithms and three stacking classifiers (i.e., meta-learners). To achieve the best prediction performance, a Bayesian optimization based multi-parameter tuning technology was adopted for the AdaBoost, random forest (RF), decision tree (DT), gradient boosting (GB) and extra tree (XTree) five machine learning models. The five parameter -tuned learners and the other four learners (i.e., logistic regression (LR), K-nearest neighbors (KNN), linear discriminant analysis (LDA), Bagging) whose parameters cannot be tuned, all as the primary week learners, were fed into the subsequent meta-learners for training and learning the final prediction model.

**Results:**

The final prediction model included 20 radiomics features and eight clinical and dosimetric characteristics. At the primary learner level, on base of Bayesian parameter tuning optimization, the RF, XGBoost, AdaBoost, GBDT, and LGBM models with the best parameter combinations achieved AUC of 0.82, 0.82, 0.77, 0.80, and 0.80 prediction performance in the verification data set, respectively. In the secondary meta-learner lever, compared with LR and MLP meta-learner, the best predictor of symptomatic RD 2+ for stacked classifiers was the GB meta-learner with an area under the curve (AUC) of 0.97 [95% CI: 0.91-1.0] and an AUC of 0.93 [95% CI: 0.87-0.97] in the training and validation datasets, respectively and the 10 top predictive characteristics were identified.

**Conclusion:**

A novel multi-region dose-gradient-based Bayesian optimization tunning integrated multi-stacking classifier framework can achieve a high-accuracy prediction of symptomatic RD 2+ in breast cancer patients than any other single deep machine learning algorithm.

## 1 Introduction

Breast cancer accounted for 11.7% of all new cancer cases and caused 685,000 deaths globally in 2020, making it the fifth leading cause of cancer death (1). Surgical interventions such as lumpectomy or mastectomy, along with radiation therapy (RT) to the residual breast or chest wall and regional lymph nodes in select cases, comprise the most common treatment for breast cancer patients (2–4). However, RT often causes acute skin toxicity, known as radiodermatitis, which manifests as erythema, scaling (dry or moist), ulceration, and necrosis. This is one of the most frequent acute side effects of RT in breast cancer patients, with erythema occurring in 90% of treated patients and wet desquamation in 30% (5–8). Radiodermatitis can negatively impact the quality of life (QOL) of breast cancer patients receiving RT in numerous ways (9). Furthermore, if radiodermatitis above grade 3 occurs, patients may need to undergo treatment withholding, radiation dose reduction, or discontinuation of treatment, which significantly increases the risk of tumor recurrence and metastasis, ultimately posing a substantial threat to the overall survival of breast cancer patients.

Acute radiation skin reactions commonly manifest during radiation therapy (RT) and pose a significant challenge to manage once they occur. However, timely intervention and management of high-risk acute radiation dermatitis can enhance patients’ daily functioning and satisfaction with radiation therapy, ultimately leading to a better quality of life and improved prognosis. Therefore, predicting the probability of acute radiation dermatitis at the outset of radiation therapy is crucial in reducing the risk of skin toxicity. Early identification of high-risk patients and prompt interventions can mitigate the severity of acute radiation dermatitis and help patients achieve better outcomes, emphasizing the significance of early prediction and management.

The qualitative assessment of acute skin toxicity through visual examination by a nurse presents numerous uncertainties such as observer bias, variability in dermatitis grade, and potential underreporting of symptoms by patients (9, 10). Furthermore, this form of assessment can only be performed after the initiation of radiotherapy, meaning that it is a reactive rather than proactive approach to treatment. As a result, routine practitioner-based visual inspection cannot be considered a reliable predictor of skin toxicity. Some researchers have attempted to improve prediction through the use of semi-quantitative analysis (11, 12) and dosimetric indicators to establish a probability model of normal tissue complications for predicting severe acute skin toxicity in breast cancer patients (13). However, the prediction performance of these methods is relatively poor, with an area under the curve (AUC) of only 0.77 (13). Despite these limitations, these approaches represent important first steps in developing more reliable predictors of skin toxicity in breast cancer patients undergoing radiotherapy, and further research in this area is warranted.

In a previous study, thermal imaging biomarkers were identified and a machine learning framework was used to build a predictive model for radiodermatitis, achieving a high predictive accuracy (test accuracy = 0.87) on the independent test data obtained at treatment fraction of 5 (10, 13). This approach advances the time of prediction to the fifth treatment fraction. However, because the prediction models only utilized the information provided by 2-D surface imaging, which limited their usage in the 3-D dose optimization guidance.

Current benchmark reports of classification algorithms for radiodermatitis prediction generally concern common classifiers, such as random forest (RF), gradient boosted decision tree (GBDT), logistic regression (LR), and XGBoost, without including the novel algorithms that have been introduced in recent years. The nine algorithms involved in this study include RF, LR, K-nearest neighbors (KNN), decision tree (DT), discriminant analysis (LDA), extra tree (XTree), gradient boosting (GB), Bagging, and AdaBoost, which have not been thoroughly investigated in existing comparative studies. Currently, the reported literatures only used limited ML methods, and the predictive performance was relatively lower, from the lowest AUC of 0.65 to the highest of 0.87 (10), which was insufficient for clinical applications. Therefore, we assume that it is difficult to obtain a clinically acceptable accuracy for radiodermatitis prediction by using a single machine learning algorithm, and an ensemble learning method could perform better than the single algorithm used as a standalone prediction tool.

Stacking is an ensemble learning technique to combine multiple classification models via a meta-classifier. The individual classification models are trained based on the complete training set; then, the meta-classifier is fitted based on the outputs ― meta-features ― of the individual classification models in the ensemble. The meta-classifier can be trained either on class labels or on probabilities from the ensemble.

In this study, we aimed to develop and verify a novel Bayesian optimization based multi-stacking deep learning platform for prediction of radiation-induced dermatitis before radiotherapy by using multi-region dose-gradient-related radiomics features extracted from the pre-treatment four-dimensional planning computed tomography (4D-CT) images, clinical and dosimetric data from breast cancer patients underwent radiotherapy. We hypothesized that acute radiodermatitis is associated with the 3D region-based characteristic radiomics signatures in breast cancer patients before RT and a well-tuned machine learner based on a multi-stacking deep learning platform can achieve better reliability and robustness of prediction performance compared to individual machine learning models.

## 2 Methods and materials

### 2.1 Patients and data collection

At three institutions including our hospital, we retrospectively collected 256 patients with breast cancer of stage 0-IV from October 2018 to August 2021 with institutional review board approval. These patients experienced post-surgery volumetric modulated arc therapy or intensity-modulated radiation therapy of prescription doses of 42.5 Gy/16 fractions or 50 Gy/25 fractions to whole breast and/or chest wall with/without boost of 10 Gy/5 fractions to the tumor bed through using the 6 MV photons, with/without concurrent hormone therapy and/or chemotherapy. With the exclusion criteria applied, including (1) loss of clinical records, (2) male patients, (3) previous skin disorder, (4) prior/subsequent RT to the chest, (5) dose boost with electron therapy, 214 patients were retained in the data analysis. All patients were graded mainly using Common Terminology Criteria for Adverse Events (CTCAE) Ver. 4, with 144 patients graded with ≥ 2 grade skin toxicity.

CT simulations of all patients were performed using the Philips Brilliance Big Bore CT scanner (Philips Medical Systems, USA) 2 to 7 days before RT with patients in breath-hold or free-breathing state. The Eclipse (Varian Medical Systems, Palo Alto, CA) or Pinnacle (Philips Medical Systems, Andover, MA) treatment planning system was utilized for the treatment planning. Clinical data, including patient characteristics, were collected alongside dose distributions and CT images for the purpose of data processing and model building.

### 2.2 Data processing

In order to extract radiomics features for our study, six regions of interest (ROIs) were delineated based on 100%, 105%, and 108% of the prescribed PTV dose, as well as 20-Gy, 30-Gy, and 40-Gy isodose of the skin. We used the Image Biomarker Explorer (IBEX) software platform to extract a total of 884 radiomics features from each ROI, which were further categorized into shape, intensity histogram, intensity direct, intensity histogram Gaussian fit, gray level run length matrix (2.5D), neighbor intensity difference (2.5D), and gray-level co-occurrence matrix (GLCM) (2.5D). Given the mild imbalanced datasets we encountered (i.e., non-RD2+ patients/total patients = 32.7%), we applied the Synthetic Minority Oversampling Technique (SMOTE) to balance all the datasets.

To select relevant clinical and dosimetric variables for further analysis, the chi-square test and the MWU test were used to calculate the P values. Variables with a P value < 0.5 were selected for subsequent steps. For the radiomics data, the MWU test (P < 0.05) was initially used, and variables with variances ≤ 0.05 were deleted as they were deemed redundant features. The pairwise correlation coefficients between the remaining variables were calculated, and variables with correlation coefficients ≥ 0.9 were removed. A variance inflation factor (VIF) was then calculated for the remaining variables, and any variables with VIF ≥ 10 were filtered out.

Because the radiation reaction of the skin is related to the fractional and total dose scheme, 123 patients in our study used the standard mode of 2Gy each fraction with a total dose of 50Gy, and other 13 patients used the moderate hypo-fractionated regimen mode of 42.5Gy in 16 daily fractions, and the remaining patients used the mode of 3~5 fractions of tumor bed electronic dose boost, so finally the EQD2_all method was adopted when performing dosimetry analysis and comparison, and the alpha/beta ratio =10 was applied for calculation of acute responding tissue to all 2Gy fractional doses.

### 2.3 Model tuning and stacking model building

Nine machine learning algorithms were used to train and validate the combined prediction models, which included clinical, dosimetric, and radiomics features. To improve the overall performance of these individual models, a stacking learning method was employed as the final decision-making strategy. Stacking is an ensemble learning technique that combines multiple classification or regression models using a meta-classifier. Unlike other ensemble methods such as Bagging or Boosting, stacking aims to decrease both bias and variance. In Bagging, similar models with high variances are averaged to decrease variance, while in Boosting, multiple incremental models are built to decrease bias while keeping variance small. In contrast, stacking uses a meta-classifier to learn how to best combine the outputs of the individual models, resulting in a more robust and accurate prediction model.

#### 2.3.1 Machine learning models comparison

In this study, nine machine learning models were utilized and compared, namely RF, LR, KNN, DT, LDA, XTree, GB, Bagging, and AdaBoost. The models underwent a thorough analysis through a 10-fold cross-validation for training purposes. To evaluate their performance, the area under curve (AUC) values were utilized as a metric for comparison. This methodology allowed for a robust analysis of the machine learning models’ effectiveness, which is important in understanding the strengths and limitations of each algorithm.

#### 2.3.2 Model tuning

In order to obtain the best training and prediction performance, a Bayesian optimization tuning method was employed to train the parameters of five algorithms, including AdaBoost, RF, DT, GB and XTree. The resulted tuning parameters were list in **Table 1**. The parameter-tuned models were fed into the subsequent multi-stacking model for ensemble training and validation.

**Table 1:** The tuning parameters for the five prediction models.

Algorithm	Tuning parameters
RF	n_estimators: 300; max_depth: [2, 20]; min_samples_leaf: 2; max_features: [0.1, 0.999]; criterion: [‘gini’, ‘entropy’]
XTree	n_estimators: 110; max_depth: 8; min_samples_leaf: 1; criterion: [‘gini’, ‘entropy’]
GB	n_estimators: 300; max_depth: 3; min_samples_leaf: 1; criterion: friedman_mse
DT	criterion: ‘gini’; max_depth: 13; min_samples_split: 1; min_samples_leaf: 2
AdaBoost	n_estimators: 120; learning_rate: 0.6

#### 2.3.3 Stacking model

The stacking ensemble method involves the creation of bootstrapped data subsets, which is similar to the bagging ensemble mechanism used to train multiple models. In this study, the nine base learners were split into two groups: those with parameter tuning, including AdaBoost, RF, DT, GB, and XTree, and those without parameter tuning, including LR, KNN, LDA, and Bagging. To determine whether the training data were properly learned, a two-layer classifier was employed. The outputs of all nine models were utilized as inputs to a meta-classifier, which predicted samples in the final step.

This two-layer classifier approach helped to ensure that the training data were accurately learned. This methodology facilitated the development of a robust ensemble model that combined the strengths of the individual models, enhancing the overall predictive performance. By leveraging the strengths of each base learner and combining them through a meta-classifier, the stacking ensemble method provided a superior predictive power.

For instance, LDA exhibits excellent performance in distinguishing between patients with and without RD2+ for certain sub-datasets (such as bootstrap data-1), whereas KNN does not perform well in predicting RD2+ for other sub-datasets (such as bootstrap data-2). The meta-classifier, which is utilized in the second layer of the stacking model, can identify and account for these discrepancies in the behaviors of the LDA and KNN classifiers, thereby improving the accuracy of the final prediction.

**Figure 1:**
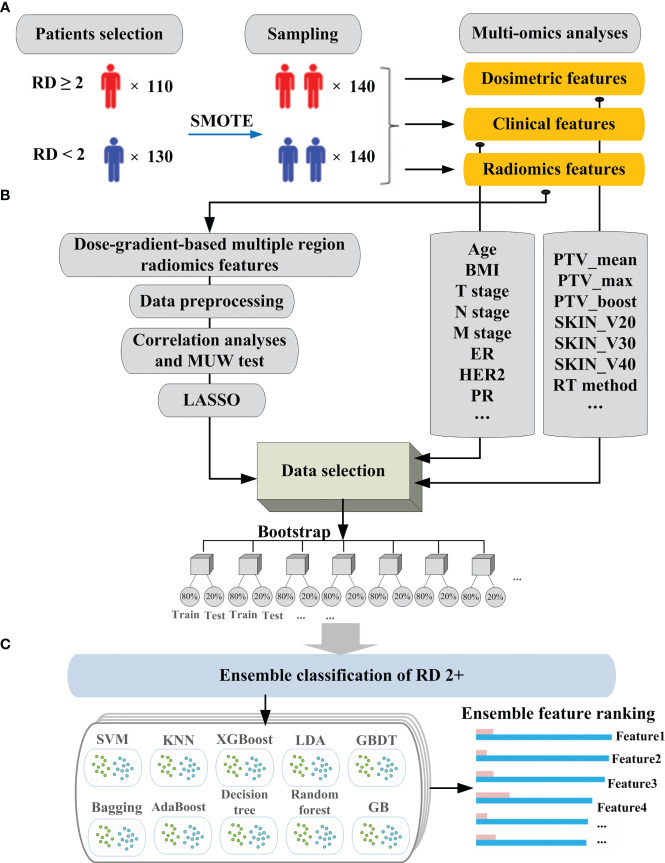
The schematic illustration of the workflow for model building in the prediction of RD 2+: **(A)** data collection and sampling; **(B)** data processing and selection; **(C)** model building and feature ranking.

**Figure 2:**
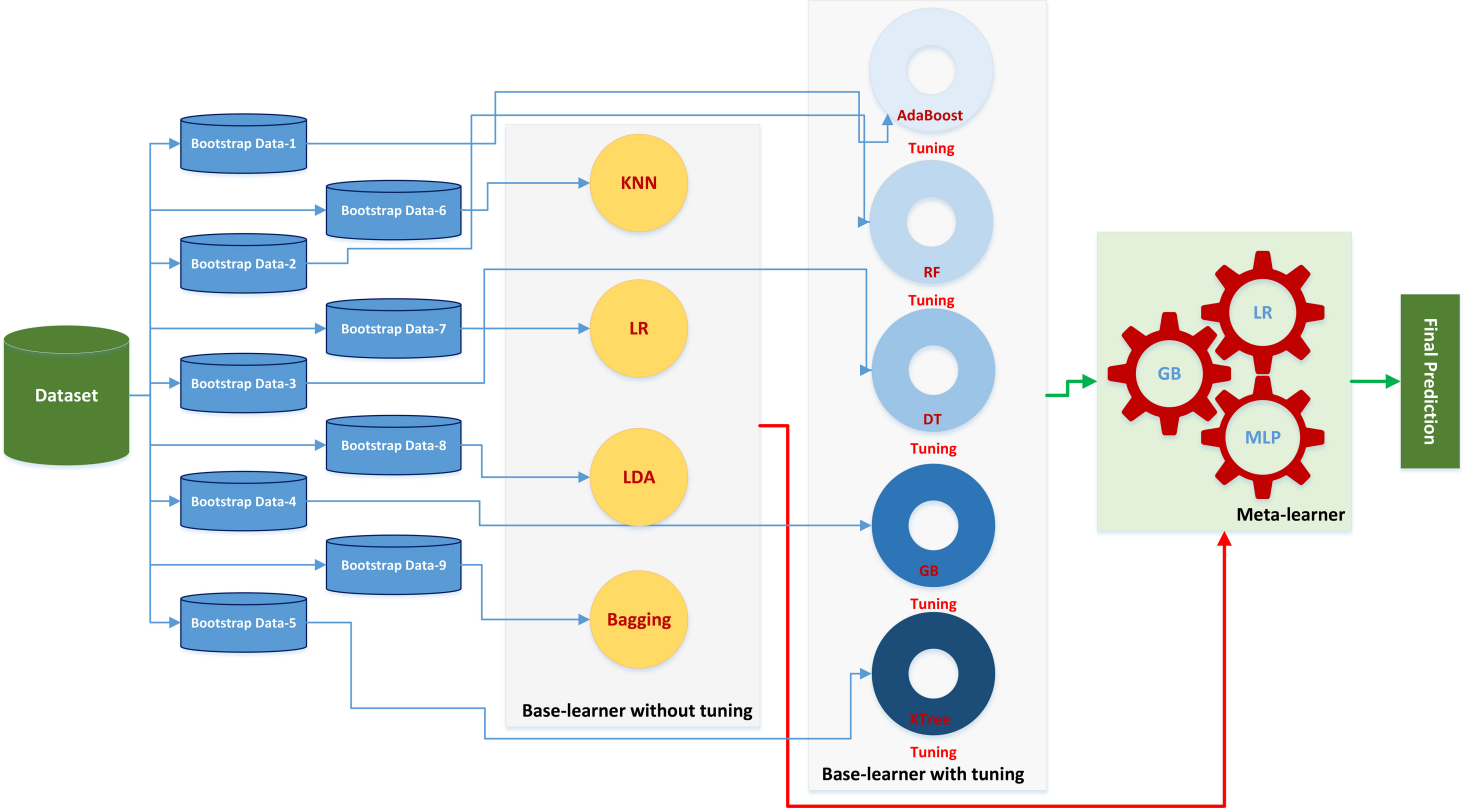
The stacking learner platform with Bayesian optimization tuned primary learner and multi-meta-learner structure.

**Figure 1** demonstrates the entire workflow of patient enrollment, extraction of clinical features and radiomics features, data cleaning and training of machine learning models. **Figure 2** illustrates the mechanism of the stacking modeling in detail, providing a visual representation of the process. By utilizing the strengths of each base learner and employing the meta-classifier to account for their different behaviors, the stacking model provides a more accurate prediction compared to any individual model.

To ensure adequate representation of RD2+ and non-RD2+ patients in the training and validation data sets, the entire data set was partitioned into ten equal sub-folds, each containing a close to 1:1 ratio of RD2+ and non-RD2+ patients without repetition. 80% of the data in each sub-fold were utilized for model training, while the remaining 20% were set aside for model validation. Implementation of the individual and multi-stacking learning algorithms was carried out using a custom Python code run in Spyder 5.5.3.

## 3 Results

There were 812, 789, 674, 684, 657, and 664 non-null features extracted from the regions of PTV_100PD, PTV_105PD, PTV_108PD, SKIN_20Gy, SKIN_30Gy, and SKIN_40Gy, respectively. The total number of samples was increased from 214 to 280 through using the resampling method of SMOTE. After applying the encapsulation screening method detailed in our previous study (8), a total of 29 clinical and dosimetric variables were selected for model building and further data analysis. These selected variables are presented in **Table 2** and were used for both training and validation in subsequent modeling processes.

**Table 2:** Final selected variables used for model training and validation.

Range	Radiomics features
PTV100PD	PTV100PD.F2._GLCM25270.7_Corr
	PTV100PD.F4.ID_LocalStdMedian
	PTV100PD.F4.ID_Range
	PTV100PD.F6.IHGaussFit1GaussMean
	PTV100PD.F8.ShapeMax3DDiameter
PTV105PD	PTV105PD.F2._GLCM25.333.7_Corr
	PTV105PD.F4.ID_LocalEntropyMax
	PTV105PD.F8.ShapeMeanBreadth
PTV108PD	PTV108PD.F1.GOH0.975Quantile
	PTV108PD.F2._GLCM25180.1Dissimilarity
	PTV108PD.F2._GLCM2590.7_IV
	PTV108PD.F8.ShapeNumberOfObjects
SKIN20Gy	SKIN20Gy.F2._GLCM25225.4Contrast
	SKIN20Gy.F8.ShapeConvexHullVolume3D
	SKIN20Gy.F8.ShapeMeanBreadth
SKIN30Gy	SKIN30Gy.F1.GOH_MAD
	SKIN30Gy.F2._GLCM25225.4Contrast
	SKIN30Gy.F4.ID_LocalRangeMax
	SKIN30Gy.F6.IHGaussFit1GaussStd
	SKIN30Gy.F8.ShapeMax3DDiameter
Clinical & dosimetric	Laterality
	Quadrant.positions.
	Histologic.type
	T.Stage
	PR
	Hormone.therapy
	Fractionation.regimen.Gy.fx.
	EQD2_all
	Lotion.application

**Figure 3:**
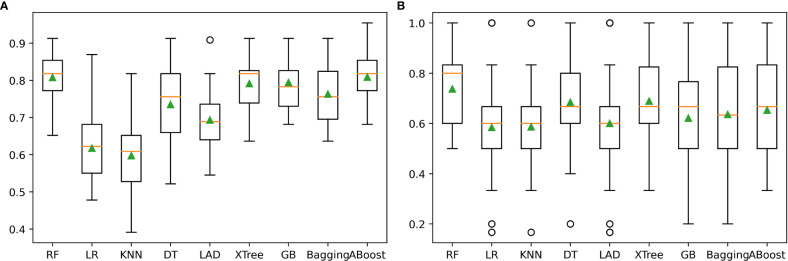
The prediction performance of nine machine learning algorithms: **(A)** illustrated the machine learner performance in training set. **(B)** illustrated the machine learner performance in validation set.

**Figure 4:**
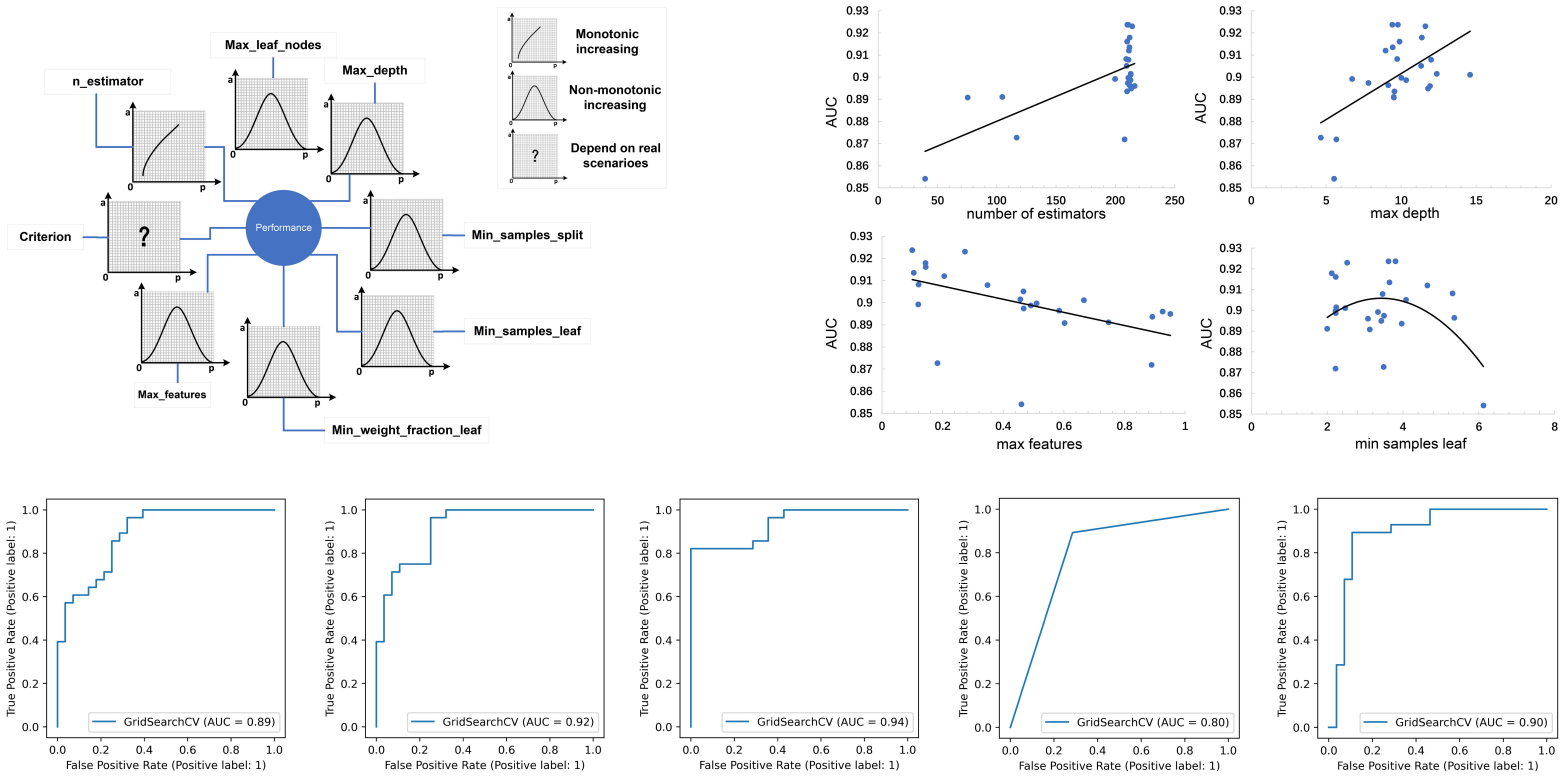
**(A)** The schematic illustration of the impacts of individual parameter settings on the prediction performance of a model, with the four parameter settings of the RF model shown as an example; **(B)** ROC curves of five tuned models in the validation set.

The performance of the nine individual models mentioned above was depicted in **Figure 3**. Notably, the RF, XTree, GB, Bagging, DT, and AdaBoost algorithms displayed impressive results in the validation set, with the highest prediction accuracy exceeding 0.8. Following parameter tuning, we identified the optimal parameter settings for the AdaBoost, RF, DT, GB, and XTree algorithms (**Table 3**), achieving the best possible prediction accuracy. Additionally, we illustrated the impact of different parameter settings on the final prediction performance of the tuned models in **Figure 4**. To evaluate the impact of Bayesian parameter optimization, we compared the performance of models with and without it in **Table 4**. We observed a substantial improvement in the prediction accuracy of the models with Bayesian parameter optimization as compared to those without it.

**Table 3:** The optimal parameter settings for the five models by using the Bayesian optimization method.

Algorithm	Optimal parameters setting
RF	criterion: gini; max_depth: 6; min_samples_leaf: 1; min_samples_split: 3; n_estimators: 200
XTree	criterion: entropy; max_depth: 8; min_samples_leaf: 1; n_estimators: 110
GB	criterion: friedman_mse; max_depth: 3; min_samples_leaf: 1; n_estimators: 300
DT	criterion: gini; max_depth: 13; min_samples_leaf: 1; min_samples_split: 2
AdaBoost	learning_rate: 0.6; n_estimators: 120

**Table 4:** Comparison of model performance with and without Bayesian parameter optimization.

Algorithm	Without Bayesian optimization tunning		With Bayesian optimization tunning	
	average AUC (training)	average AUC (validation)	average AUC (training)	average AUC (validation)
RF	0.808	0.738	1.0	0.891
XTree	0.791	0.689	0.980	0.923
GB*	0.795	0.621	1.0	0.943
DT	0.735	0.684	1.0	0.801
AdaBoost	0.809	0.653	1.0	0.903

* Gradient boosting(GB).

**Figure 5:**
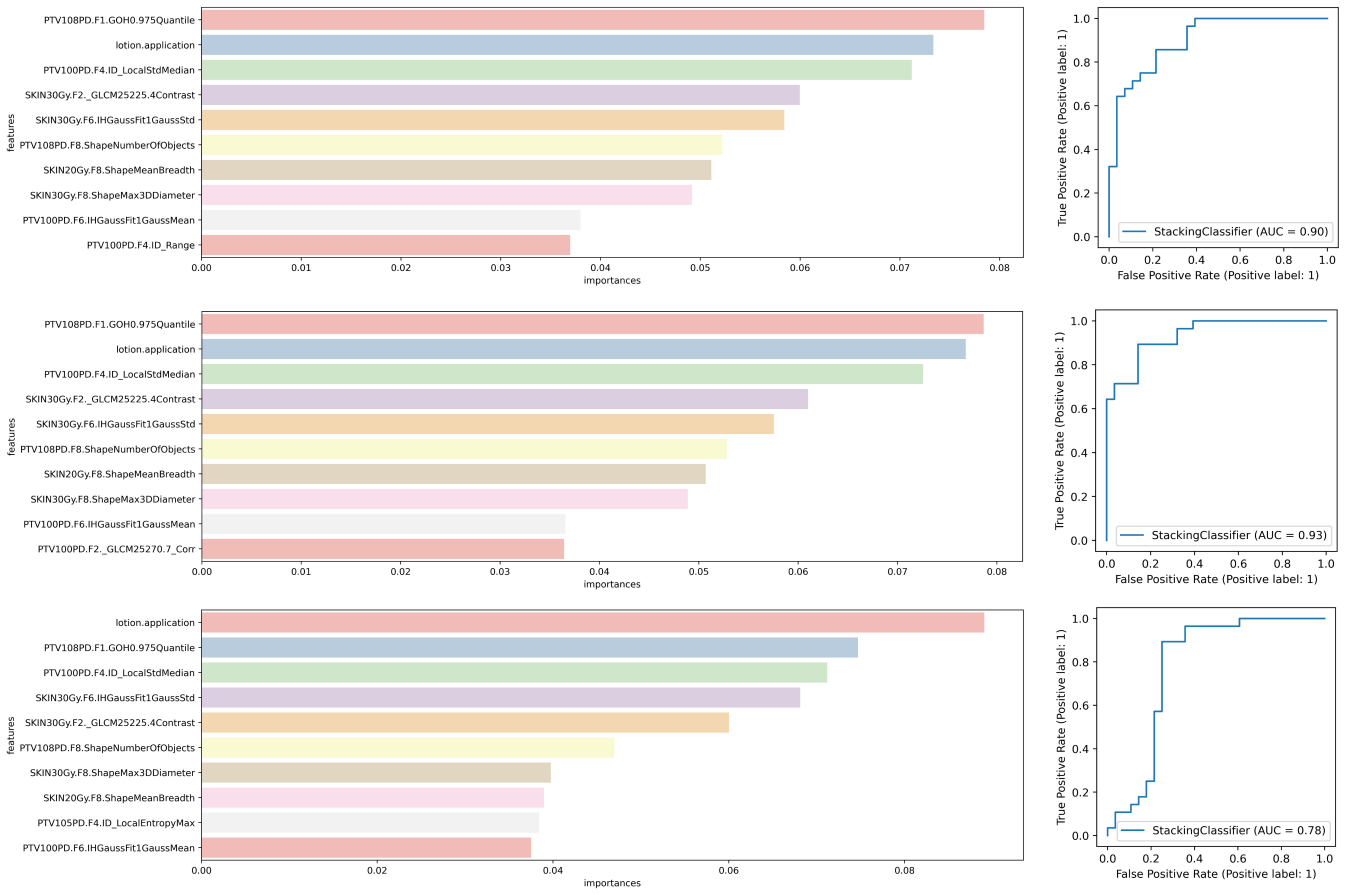
The prediction performance of the stacking model with three different meta -learners and variable importance ranking.

**Figure 2** illustrates that the base learner was formed by combining five tuned classifiers and four other prediction models without tuning. For the stacking model, we selected three meta-learners ― LR, GB, and MLP. The MLP comprised two hidden layers, each with 16 neurons. We evaluated the final prediction performances of the stacking models with the three different meta-learners and ranked the variable importance accordingly, as depicted in **Figure 5**.

## 4 Discussion

The ensemble learning model leverages a series of weak learners, also known as base learners, to improve learning performance by combining the results of each weak learner (9). Common algorithms for integrated learning models include aggregation algorithm (e.g., Bagging), boosting algorithm (e.g., Boosting) and stacking method (e.g., Stacking). The random forest model is a typical machine learning model using the Bagging algorithm, while the AdaBoost, GBDT, XGBoost, and LightGBM models are typical machine learning models using the Boosting algorithm.

Only one abstract and two full papers were found that focused on using machine learning methods for predicting radiodermatitis. Saednia et al. (6) conducted a study based on detecting an increase in body surface temperature induced by radiation dermatitis. They built a random forest classifier using a leave-one-out cross-validation approach to predict radiodermatitis, achieving an accuracy of 0.87 in an independent test dataset. However, detailed information regarding the random forest classifier was not provided. For instance, the authors did not explain the rationale behind choosing the random forest over other machine learning algorithms or provide clear explanations of the parameter configurations, such as max features, maximum depth of the decision tree, the minimum number of samples required for internal node subdivision, and the minimum number of samples in leaf nodes.

it will be interesting and helpful to specify the existing data related to DVH, NTCP models and the value and limits of predictive dosimetric models, however, the above-mentioned method of predicting radiation dermatitis through DVH parameters, NTCP models or dosimetry parameters may be a relatively traditional prediction method. A researcher from Italy, Giuseppe Palma (14) who found that NTCP models showed comparable high prediction and calibration performances with a balanced accuracy of 0.76 with LKB and 0.78 with multi-parameter Logistic respectively, and our final model with an area under the curve (AUC) of 0.97 [95% CI: 0.91-1.0] and an AUC of 0.93 [95% CI: 0.87-0.97] in the training and validation datasets.

In another study, 2277 patients were included to predict acute toxicities during radiation therapy for breast cancer patients using three machine learning models (RF, GBDT, LR), with AUC values for RD2+ of 0.807, 0.811, and 0.813, respectively (4) It is well -known that different training parameter settings have a significant impact on the predictive ability of the machine learning model (5). However, the study did not provide a detailed discussion on the specific parameter settings of the machine learning training model.

The results of our study indicate (**Figure 4**) that increasing the value of n_estimators improved the model’s performance. A larger value of n_estimators indicates that more subtrees are involved in the decision-making process, which can eliminate random errors between subtrees, increase prediction accuracy, and reduce both variance and bias. However, if n_estimators is too small, underfitting may occur, and if it is too large, the model may not be significantly improved. Therefore, a moderate value of n_estimators should be chosen. In the case of increasing the value of max_depth or max_leaf_nodes, prediction accuracy typically increases initially, followed by a decrease. This decrease occurs as subtree complexity increases, which reduces deviation but increases variance. Thus, optimization of these parameter settings significantly affects prediction results, and the conclusions of the study should be based on this information.

In the cited studies, important aspects of the model training, such as the use of out-of-bag samples for model evaluation and the feature evaluation criteria, were not discussed. Additionally, it is important to note that classification and regression models differ in their default loss functions: the classification model typically utilizes the Gini index or information gain, while the regression model utilizes the mean square error (MSE) or mean absolute error (MAE). Unfortunately, the specific loss function used in the models was not clearly reported in these studies. Understanding these details is crucial to accurately interpret the results of machine learning models and ensure reproducibility.

After conducting a thorough examination of previous studies on the prediction of radiation injury using various machine learning models, we discovered that the selection of machine learning algorithms and the setting of specific training parameters varied widely among research institutions. In some cases, the default parameter settings of the algorithm were used in model training without any parameter optimizations. However, according to a study conducted by Zhang et al, it was found that GBDT and RF demonstrated the best overall classification accuracy and mean rank across all 71 data sets, suggesting that these algorithms are optimal for different data structures and classification tasks. It is likely for this reason that all the aforementioned studies utilized RF as their chosen machine learning algorithm.

ML algorithms each have their own hyperparameters, and good performance can only be achieved when appropriate hyperparameters are set in the learning process. It is very tedious to manually adjust proper parameters, and it is not easy to optimize the parameters for the best performance. In general, when creating an ensemble model, searching methods such as grid search, random search and Bayesian optimization are used for tuning the hyperparameters of each base model.

Bayesian optimization tries to gather observations with the highest information in each iteration by striking a balance between exploring uncertain hyperparameters and gathering observations from hyperparameters close to the optimum (15, 16). Therefore, in the stage of training the base learner of stacking ensemble learning, if the hyperparameters of individual models can be optimized using Bayesian optimization, the accuracy of the model can be increased.

The impact of model parameter tuning on the final predictive ability of the model is a common issue that is often overlooked in many studies. This study addresses this problem by utilizing Bayesian optimization parameter tuning to determine the most suitable parameter settings for optimal performance of each model. **Table 3** indicates that the AdaBoost algorithm achieves better results when the learning rate is relatively small (such as 0.6) with an n_estimators value of 120. Additionally, **Figure 4** shows that the AUC values of the RF model in the test set almost monotonously increase with the increase of n_estimators and max depth, whereas the AUC value monotonically decreases with the increase of max features. It is also observed that the AUC of the RF model increases at first and then decreases with the increase of min sample leaf. These results suggest that the adjustment and optimization of parameters is necessary for specific machine learning models. Selecting the most suitable algorithm with corresponding optimized parameter settings can lead to the best predictive performance of the machine learning model.

Although we utilized the Bayesian optimization parameter tuning method in our study, it is important to note that the optimal value obtained may not necessarily be the global optimal value. Instead, it may be a local optimal value. Moreover, since Bayesian optimization is based solely on the training dataset, overfitting of the model can occur. Therefore, developers must be cautious and strive to reduce the boundary settings of overfitting parameters. Furthermore, it is worth mentioning that simply tuning parameters to create a slightly better model does not necessarily result in a significant improvement in the final results. In fact, great improvements can be achieved through the use of feature engineering and model integration methods.

**Figure 2** illustrates a one-level meta-classifier stacking model, which is a popular method used to improve model performance. However, multi-level stacking ensemble methods are also employed, wherein additional layers of classifiers are added to the stacking model. In this study, we fed four classifiers without parameter tuning and five other prediction models with Bayesian parameter tuning into a single-level meta-classifier, employing LR, GB, and MLP as the three meta-classifiers. Although this approach can improve model performance, it can become computationally expensive for only a small boost in performance. As such, researchers must balance the trade-off between computational cost and model performance and carefully choose the appropriate method for their specific application.

The stacking learner is a popular integrated learning method widely used by many researchers due to its strong flexibility and outstanding prediction performance, despite receiving less support by theoretical research in mathematics compared to boosting and bagging (4, 5). However, selecting the appropriate machine learning type for both the base learner and the meta-learner remains a longstanding and unresolved issue. Some studies suggest using the same machine learner for both the base learner and secondary learner, while others propose using simple learners as base learners and more complex learners as meta-learners. In these cases, the stacking performance seems to be more robust in terms of classification accuracy (6, 7). Therefore, the choice of the machine learning types for both base and meta-learners should be carefully considered to obtain optimal stacking performance.

Mohanad Mohammed proposed a novel stacking ensemble deep learning model based on a one-dimensional convolutional neural network (1D-CNN) to perform multi-class classification on five common cancers among women using RNASeq data. The stacking ensemble method comprised support vector machines with linear and polynomial kernels, artificial neural networks, KNN, and bagging trees, resulting in a sound prediction performance with a mean accuracy of 0.99. Liang M. developed a stacking ensemble learning framework (SELF) by integrating three machine learning methods to predict genomic estimated breeding values (GEBVs), achieving a high prediction accuracy of 0.855. Y. Xiong et al. reported an ensemble learning platform that combined a primary learner stack, including a library for support vector machine (LIBSVM), KNN, DT (C4.5), and RT, as the primary learners of the stacking ensemble. In their work, the embedding cost-sensitive naive Bayes was utilized as the meta-learner of the stacking ensemble, resulting in accuracy ranging from the minimum value of 87.93% to the maximum value of 100%. Overall, these studies demonstrate the effectiveness of the stacking ensemble method in different applications and highlight its potential for improving prediction performance in various fields.

Previous studies suggest that integrating a traditional weak learner as the base learner with a more complex meta-learner can improve the prediction performance of the model. In our work, we utilized three different learners as meta-learners and evaluated their performance. Through experiments, we found that the GB learner was the most suitable meta-learner, achieving the highest AUC value compared to the other two meta-learners. This finding suggests that careful selection of meta-learners can enhance the reliability and robustness of the final output of the model. Therefore, choosing an appropriate meta-learner is crucial to ensure the effectiveness of the stacking ensemble method in improving prediction performance.

## 5 Conclusion

In conclusion, we have proposed a novel multi-region dose-gradient-based multi-stacking classifier framework that incorporates Bayesian optimization parameter tuning to achieve an ultra-high accuracy prediction of symptomatic RD 2+ in breast cancer patients who underwent radiotherapy. Our approach represents a significant improvement over traditional models and has the potential to improve the clinical management of breast cancer patients by enabling early identification of those at risk of developing radiation-induced toxicity. With further validation and refinement, our framework may have broader applications in other radiation therapy contexts, ultimately improving the accuracy and efficacy of cancer treatments.

## Data availability statement

The raw data supporting the conclusions of this article will be made available by the authors, without undue reservation.

## Ethics statement

The studies involving human participants were reviewed and approved by Ethical Review Approval Document of the Ethics Committee of Hangzhou Cancer Hospital-HZCH-2022. The patients/participants provided their written informed consent to participate in this study.

## Author contributions

XL created the study design. KW collected the clinical and computed tomography data and processed the data. KW, XM conducted data analysis. XL and KW wrote the manuscript. HW gave suggestions regarding the radio-dermatitis grading. All authors contributed to the article and approved the submitted version.
